# A rare case of chronic hypersensitivity pneumonitis with pleural lipoma

**DOI:** 10.11604/pamj.2024.47.187.42917

**Published:** 2024-04-16

**Authors:** Ashwin Karnan, Bollineni Sreeprada

**Affiliations:** 1 Department of Respiratory Medicine, Datta Meghe Institute of Higher Education and Research, Sawangi (Meghe), Wardha, Maharashtra, India

**Keywords:** Hypersensitivity, steroids, dyspnea, lipoma, inflammation

## Image in medicine

A 72-year-old female presented with complaints of dry cough, breathlessness, and mild chest pain, on and off for the past 2 years. She had no comorbidities, no significant past or personal history, and gave a history of having 3 pet pigeons at home for the past 3 years. Chest X-ray was unremarkable. The computed tomography of the chest showed bilateral ground glass opacities with mosaic attenuation predominantly in the upper lobes suggestive of hypersensitivity pneumonitis with a hypodense lesion (-90HU) in the pleural space suggestive of pleural lipoma. Serum pigeon-specific IgG was elevated. The patient was started on oral prednisolone 1mg/kg and advised to avoid exposure to pigeons. At 2 month follow up there was remarkable improvement in symptoms. Pleural lipomas are the most common benign soft tissue tumor of the pleura. They originate from the parietal pleura and may extend into the subpleural, pleural, or extra-pleural space. They are usually asymptomatic but, in some cases, may cause pleural irritation and cough. CT scan is the investigation of choice. Any rounded tumor, originating from the parietal or mediastinal pleura, homogenous, without any calcification, with an adipose density of -50 to -150 HU and not enhanced by an injected contrast medium, is a lipoma. Pleural lipomas usually need no treatment other than observation but in rare cases of large tumors, video-assisted thoracoscopic surgery may be needed.

**Figure 1:**
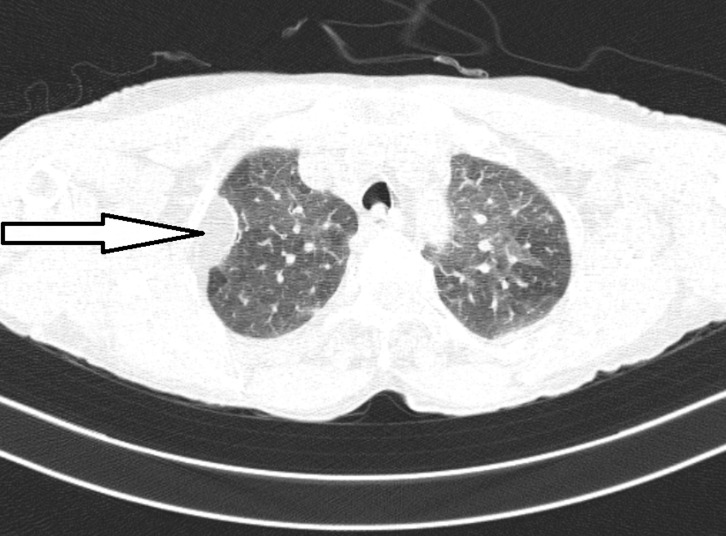
computed tomography showing bilateral ground glass opacities with mosaic attenuation with a white arrow showing homogenous hypodense (-90HU) lesion in pleural space suggestive of pleural lipoma

